# Achilles tendon suture deteriorates tendon capillary blood flow with sustained tissue oxygen saturation – an animal study

**DOI:** 10.1186/1749-799X-4-32

**Published:** 2009-08-12

**Authors:** Robert Kraemer, Johan Lorenzen, Robert Rotter, Peter M Vogt, Karsten Knobloch

**Affiliations:** 1 Plastic, Hand and Reconstructive Surgery, Hannover Medical School, Carl-Neuberg-Strasse 1, 30625 Hannover, Germany; 2 Department of Nephrology, Hannover Medical School, Carl-Neuberg-Strasse 1, 30625 Hannover, Germany; 3 Department of Trauma and Reconstructive Surgery, University of Rostock, Schillingallee 35, 18057 Rostock, Germany

## Abstract

**Background:**

Treatment of ruptured Achilles tendons currently constitutes of conservative early functional treatment or surgical treatment either by open or minimal invasive techniques. We hypothesize that an experimental Achilles tendon suture in an animal model significantly deteriorates Achilles tendon microcirculation immediately following suturing.

**Methods:**

Fifteen Achilles tendons of eight male Wistar rats (275–325 g) were included. After preparation of the Achilles tendon with a medial paratendinous approach, Achilles tendon microcirculation was assessed using combined Laser-Doppler and spectrophotometry (Oxygen-to-see) regarding:

- tendinous capillary blood flow [arbitrary units AU]

- tendinous tissue oxygen saturation [%]

- tendinous venous filling pressure [rAU]

The main body of the Achilles tendon was measured in the center of the suture with 50 Hz. 10 minutes after Achilles tendon suture (6-0 Prolene), a second assessment of microcirculatory parameters was performed.

**Results:**

Achilles tendon capillary blood flow decreased by 57% following the suture (70 ± 30 AU vs. 31 ± 16 AU; p < 0.001). Tendinous tissue oxygen saturation remained at the same level before and after suture (78 ± 17% vs. 77 ± 22%; p = 0.904). Tendinous venous filling pressure increased by 33% (54 ± 16 AU vs. 72 ± 20 AU; p = 0.019) after suture.

**Conclusion:**

Achilles tendon suture in anaesthetised rats causes an acute loss of capillary perfusion and increases postcapillary venous filling pressures indicating venous stasis. The primary hypothesis of this study was confirmed. In contrast, tendinous tissue oxygen saturation remains unchanged excluding acute intratendinous hypoxia within the first 10 minutes after suture. Further changes of oxygen saturation remain unclear. Furthermore, it remains to be determined to what extent reduced capillary blood flow as well as increased postcapillary stasis might influence tendon healing from a microcirculatory point of view in this animal setting.

## Background

Achilles tendon ruptures are currently treated either conservatively with an early functional rehabilitation programme or surgically with varying suture techniques. Besides the conventional open approach with its known inherited potential adverse effects such as infections and extensive scaring minimal-invasive percutaneous techniques are becoming popular. Current scientific studies adress biomechanical properties of different suture techniques for tendon ruptures. [1-3] Furthermore, varying suture techniques might effect the tendon in different ways. [4] Microcirculation of both healthy and tendinopathic Achilles tendons has been assessed, whereas tendinopathic tendons showed an altered microcirculation with increased capillary blood flow at the point of pain. [5] Despite the various reports of the biomechanical properties of both healthy and torn Achilles tendons, tendon microcirculation following tendon suture neither in humans nor in an experimental animal setting has been determined yet.

As tendon healing is often prolonged and associated with a less than optimal outcome, current studies aim at ameliorating the conditions for tendon healing. [6,7] However, the underlying mechanisms still remain largely unknown. Currently, we do not know whether the tendon suture might effect or even deteriorate tendon microcirculation by a potential compression of the tendon vasculature.

We therefore designed a preliminary animal study to investigate our hypothesis that an Achilles tendon suture significantly deteriorates Achilles tendon microcirculation immediately following the tendon suture.

## Methods

Experiments were approved by the local animal right protection authorities and performed in accordance to the NIH guidelines for the Care and Use of Laboratory Animals (Institute of Laboratory Animal Resources, National Research Council).

### Experimental Protocol

The study included 8 male Wistar rats (250–350 g body weight (bw); Charles River Laboratories, Sulzfeld, Germany), housed 1/cage at 21°C with a natural light/dark cycle as well as water and standard laboratory chow ad libitum. Under pentobarbital sodium anesthesia (55 mg/kg bw ip; Narcoren, Merial, Hallbergmoos, Germany), the rats were tracheotomized and mechanically ventilated (tidal volume 1 ml/100 g bw; 50 breaths/min). Furthermore, animals' right carotid artery was cannulated with polyethylene catheters (PE 50, 0.58 mm inner diameter; Portex, Hythe, Kent, UK). The cannulation allowed heart rate and blood pressure to be monitored. During the procedure, body temperature was maintained at 36–37°C by means of a heating pad. Systemic blood parameters and blood gas analysis were assessed before starting measurements using a Coulter Counter (AcTdiff, Coulter, Hamburg, Germany).

Fifteen gastrocnemius and soleus muscles as well as Achilles tendons were surgically exposed by a medial approach down to the calcaneal insertion. Only 15 Achilles tendons of 8 Wistar rats could be used because of carotid cannulation dislocation with consecutive fatal bleeding and exitus letalis after measurement of a single tendon in one case.

Tissue was allowed to stabilize for 10 minutes before investigating the tendinous microcirculation using a non-invasive combined Laser-Doppler- and Photospectrometry-system (Oxygen-to-see, O2C, LEA Medizintechnik, Giessen, Germany). Each tendon was measured 10 minutes after preparation of the Achilles tendon as well as ten minutes after tendon suture at the same location. Measurements at baseline and 10 minutes after tendon suture always started at the right Achilles tendon. 15 minutes after initiation of measurement of the right Achilles tendon of one rat, the contralateral Achilles tendon was measured at baseline.

**Figure 1:**
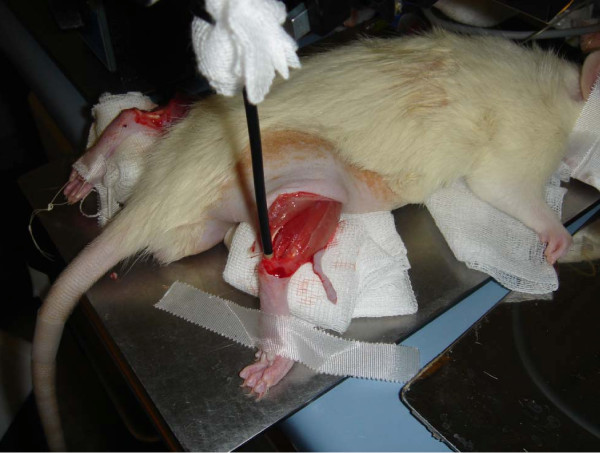
**Baseline measurements with Oxygen-to-see micro-probe for real-time microcirculatory assessment of capillary blood flow, tendon oxygen saturation and postcapillary venous filling pressure after preparation of both Achilles tendons**.

We used a fixation apparatus for the probe in order to minimize measurement artefacts due to vibration (figure 1). Baseline measurements were carried out on the Achilles tendon for one minute on each tendon's mid-portion 4 mm proximal of the Tuber calcanei before tendon suture. We applied a tendon frame suture in the means of a Kirchmayer's suture at the same distinct location on the tendon's mid-portion with Prolene 6-0 BV (11 mm 3/8 c, Ethicon, USA). Ten minutes after suturing with knotting, the microcirculatory data was assessed again for one minute on the same location on each site.

### Microcirculatory Analysis

The determination of hemoglobin and the principle of blood flow measurement are combined in the O2C system. The optical method for measuring both, blood flow by Laser-Doppler technique and hemoglobin oxygenation and hemoglobin concentration in tissue by spectrometric techniques, has been described in detail elsewhere. [8] The local oxygen supply parameters, blood flow, oxygen saturation of hemoglobin, and the relative postcapillary venous filling pressures were recorded by an optical fiber probe. The fiber probe incorporates both the laser Doppler method and the broadband light spectrometry technique. The probe we used assessed data in 200 micrometer depth regarding:

• tendinous capillary blood flow [arbitrary units; AU]

• tendinous tissue oxygen saturation [%]

• tendinous venous filling pressure [AU]

We have recently described the use of the O2C-system in measurements of increased capillary blood flow at the point of pain in patients with insertional and mid-portion tendinopathy of the Achilles tendon compared to healthy subjects as well as the influence of permanent and intermittent application of cooling and compression on microcirculation of Achilles tendons in healthy humans. [5,9] A 5% intrasubject variability was determined for the Oxygen-to-see system indicating that a laser Doppler is a reliable method under sufficient standardized test conditions. [10]

### Statistical Analysis

Independent samples t-test was applied for comparison of baseline measurement of right vs. left Achilles tendon. Paired t-test was used for comparison of pre- vs. post-interventional microcirculatory changes. A p-value less than 0.05 was considered to indicate statistical significance. The SPSS statistical software package 16.0 for Windows (SPSS Inc., Chicago, Ill, USA) was used for statistical analysis.

## Results

### Microcirculatory Achilles tendon blood flow

**Figure 2:**
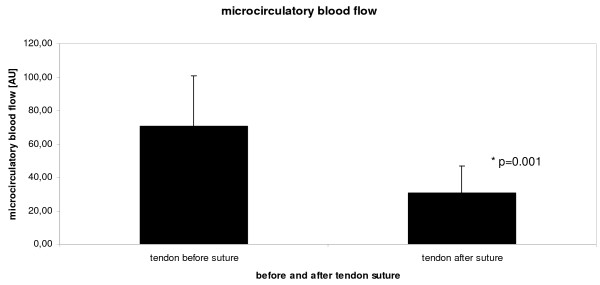
**Achilles tendon capillary blood flow [arbitrary units, AU] before (left column) and ten minutes after (right column) tendon suture with 6-0 prolene in anesthetized rats**.

Achilles tendon mid-portion capillary blood flow was 70 ± 30 arbitrary units at rest without tendon suture. There were no significant differences in capillary blood flow between right and left Achilles tendon at baseline (70 ± 41 vs. 71 ± 17 AU; p = 0.952). 10 minutes after suturing the tendon's mid portion, capillary blood flow significantly decreased to 31 ± 16 arbitrary units by 57% (p < 0.001, figure 2).

### Achilles tendon oxygen saturation

**Figure 3:**
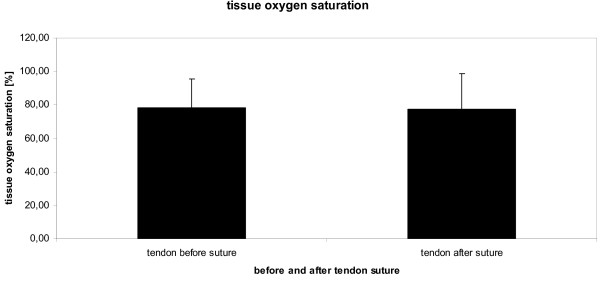
**Achilles tendon oxygen saturation [%] before (left column) and ten minutes after (right column) tendon suture with 6-0 prolene in anesthetized rats**.

Achilles tendon mid-portion oxygen saturation was 78 ± 17% at rest without tendon suture. There were no significant differences in oxygen saturation between right and left Achilles tendon at baseline (74 ± 21 vs. 84 ± 13 AU; p = 0.340). Oxygen saturation did not significantly change within ten minutes following the suture of the Achilles tendon and remained at 77 ± 22% (p = 0.902, figure 3).

### Achilles tendon relative postcapillary venous filling pressure

**Figure 4:**
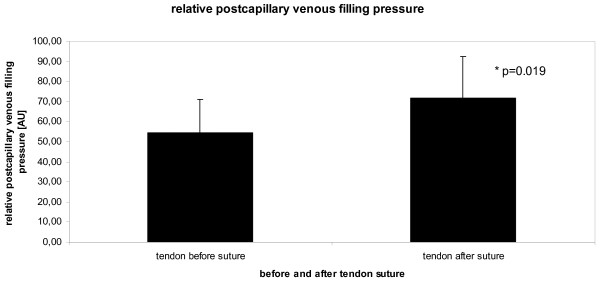
**Achilles tendon relative postcapillary venous filling pressure [arbitrary units, AU] before (left column) and ten minutes after (right column) tendon suture with 6-0 prolene in anesthetized rats**.

Achilles tendon postcapillary venous filling pressure was 54 ± 16 arbitrary units at rest without tendon suture. There were no significant differences in postcapillary venous filling pressure between right and left Achilles tendon at baseline (56 ± 15 vs. 53 ± 20 AU; p = 0.609). The postcapillary filling pressure significantly increased ten minutes after the Achilles tendon suture to 72 ± 20 AU (p = 0.022, figure 4).

## Discussion

The main findings of our study are: Achilles tendon suture in anaesthetised rats causes an acute loss of capillary perfusion and increases postcapillary venous filling pressures indicating venous stasis. The primary hypothesis of this study was confirmed. In contrast, tendinous tissue oxygen saturation remains unchanged excluding acute intratendinous hypoxia within 10 minutes after suture.

Current scientific data demonstrates a rapid ingrowth of new vessels and nerve fibers, expressing sensory and autonomic neuropeptides, in the healing tendon after injury. As the tendon heals, nerves and vessels withdraw. [11,12] The three consecutive, overlapping phases of tendon healing, that is, the inflammatory, proliferative and remodelling phase, exhibited clear differences in collagen formation and neuronal occurrence. The first signs of newly organized collagen can be noted during the inflammatory phase in week 2 after tendon rupture. [13] While current scientific data are mainly focussing histological investigation of tendon healing phases, our study aimed at determination of the microcirculatory in vivo effects in an animal model immediately after Achilles tendon suture during the early inflammatory phase.

However, as clinical data on this issue are pending, a limitation of our study obviously is that animal data neither correspond to a human tendon nor to a clinical situation. Furthermore, different suture types as well as knot techniques might change the Achilles tendon microcirculation differentially, which has to be taken into account when interpreting our data. In our study a Kirchmeyer's suture was used for Achilles tendon suture as a common suture technique in Achilles tendon rupture in humans. We studied healthy and structural intact Achilles tendons of rats. In a torn Achilles tendon the microcirculatory situation might be different.

We used a Kirchmeyer's suture for Achilles tendon suture as a commonly used tendon suture technique in an open approach with similar biomechanical properties compared to the Bunnell technique. [14] However, given the frame suture line applied in our preliminary study, the current suture lines are suspected to cause even more compression to the center of the tendon since various suture lines isolate the tendon center. Our study aims at evaluation of tendinous microcirculatory data in an animal model, but further animal studies are necessary to elucidate the various suture lines with their potential impact on tendon microcirculation at least in preliminary animal settings.

In our animal study, Achilles tendon suture in anaesthetised rats causes a markedly reduced capillary perfusion and increased postcapillary venous filling pressures indicating venous stasis within ten minutes after tendon suture. The primary hypothesis of this study was confirmed. However, tendinous tissue oxygen saturation remains unchanged excluding acute intratendinous hypoxia within 10 minutes after suture. These data indicate that tendon ischemia was not encountered at least within 10 minutes after a conventional suture in a rat model. Beyond this time frame we currently do not know whether tendon oxygenation might deteriorate subsequent to the reduced capillary inflow or the venous congestion. Current anatomic research shows an Achilles tendon perfusion from the ventral paratendinous tissue which implies sustained tissue oxygen saturation even over a longer time period. To what extent paratendinous perfusion is involved in ameliorating Achilles tendon microcirculation is speculative. Nevertheless, reduced capillary blood flow as well as increased postcapillary stasis might influence tendon healing from a microcirculatory point of view at least in this preliminary animal experiment.

For further studies we hypothesize that microcirculation could normalize in regularly healing animal tendons. A recent clinical study demonstrated deep venous thrombosis in up to 34% of patients treated either conservatively or operatively after Achilles tendon rupture. [15] In these cases, postcapillary stasis could be apparent during the whole tendon healing process which indeed might constrain or limit tendon healing.

Recently, it was hypothesized that Intermittent Pneumatic Compression (IPC) may exert positive effects on tissue healing, a process highly dependent upon adequate circulation. IPC is a treatment based on the passive increase of blood flow by cycling external pressure. The biomechanical effects include reduced venous stasis, decreased venous pressure and increased arterial blood flow. [16] IPC is used to prevent thrombosis in immobilized patients, though the circulatory increase has also been hypothesized to benefit tissue healing. [17] IPC also promotes fracture healing by enhancing callus formation and biomechanical strength. [18,19] In a recent animal study, the effects of a daily 1-h IPC treatment during 2 and 4 weeks after rat Achilles tendon rupture were investigated. The daily IPC treatment improved neurovascular ingrowth and fibroblast proliferation in the healing tendon and may therefore accelerate the repair process. [20] As we found an increased venous stasis right after tendon suture with a decreased arterial blood flow in our animal model, IPC should also be investigated about enhancing the healing process after Achilles tendon suture at least in an experimental animal setting.

Furthermore, in vivo transient limb ischemia releases a low molecular weight, hydrophobic, circulating factor which induce a potent protection against myocardial ischemia/reperfusion injury in Langendorff perfused hearts and isolated cardiomyocytes in the same species. This cardioprotection is transferable across species, independent of local neurogenic activity, and requires opioid receptor activation.[21] A commonly used temporary intraoperative tourniquet should therefore not compromise local healing of the sutured tendon and furthermore could even ameliorate the tendon microcirculation leading to a better tendon healing.

## Conclusion

Achilles tendon suture in anaesthetised rats causes an acute loss of capillary perfusion and increases postcapillary venous filling pressures indicating venous stasis. In contrast, tendinous tissue oxygen saturation remains unchanged excluding acute intratendinous hypoxia within 10 minutes after suture. Reduced capillary blood flow as well as increased postcapillary stasis might influence tendon healing from a microcirculatory point of view at least in this animal experiment. Nevertheless, the consequences of our findings remain unclear and must be an issue of further studies.

## Ethical statement

Ethical Board Review statement: Experiments were approved by the local animal right protection authorities and performed in accordance to the NIH guidelines for the Care and Use of Laboratory Animals (Institute of Laboratory Animal Resources, National Research Council).

## Competing interests

The authors declare that they have no competing interests.

## Authors' contributions

RK carried out the data assessment. RR performed preparation of the tendons and supplied materials. RK, JL and KK drafted the manuscript. RK and KK participated in the design of the study. RK and JL performed the statistical analysis. PMV conceived of the study, participated in its design and coordination and helped to draft the manuscript. All authors read and approved the final manuscript.
